# Mercury bioremoval by *Yarrowia* strains isolated from sediments of mercury-polluted estuarine water

**DOI:** 10.1007/s00253-014-6279-1

**Published:** 2014-12-18

**Authors:** Ganiyu Oladunjoye Oyetibo, Shakirat Titilayo Ishola, Wakako Ikeda-Ohtsubo, Keisuke Miyauchi, Matthew Olusoji Ilori, Ginro Endo

**Affiliations:** 1 Department of Civil and Environmental Engineering, Faculty of Engineering, Tohoku Gakuin University, 1-13-1 Chuo, Tagajo, Miyagi 985-8537 Japan; 2 Department of Microbiology, Faculty of Science, University of Lagos, Akoka, Yaba, Lagos Nigeria

**Keywords:** Mercury-resistant yeast, Resting and growing cells, Mercury bioremoval, Biosorption, Biovolatilization

## Abstract

Difference in mercuric ion removal by resting and growing cells of two mercury-resistant yeast strains, identified as *Yarrowia* spp. (strains Idd1 and Idd2), were studied. Resting cells of strain Idd2 exhibited high maximum Hg^2+^ removal capacity (59 mg mercury per g dry cell weight [gdw^−1^]) by adsorption than those of resting cells of strain Idd1 (32 mg gdw^−1^). The resting cells of strain Idd2 exhibited a higher Hg^2+^ desorption capacity using CaCl_2_ (68 %) and EDTA (48 %) than strain Idd1, depicting weaker binding of Hg^2+^ onto strain Idd2 unlike strain Idd1. The actively growing yeast cells showed opposite Hg removal characteristics to those of the resting cells. Strain Idd1 adsorbed less Hg^2+^ from culture medium supplemented with Hg^2+^ than strain Idd2. However, the growing strain Idd1 reduced and vaporized 27 % of supplemented Hg^2+^ as metallic mercury (Hg^0^), while the growing strains Idd2 vaporized 15 % of the supplemented Hg^2+^. These two yeast strains are potential biotechnological tools for the eventual bioremediation of polluted aquatic systems.

**Electronic supplementary material:**

The online version of this article (doi:10.1007/s00253-014-6279-1) contains supplementary material, which is available to authorized users.

## Introduction

Mercury is a major pollutant and the most widely distributed toxic metal in the environment (Tchounwon et al. 2003). Natural sources are the major means through which Hg is introduced into the environment (Tomiyasu et al. 2006; 2008). However, anthropogenic activities remain an unparalleled channel through which various species of Hg polluted the biosphere (AMAP/UNEP 2013; Nimick et al. 2013; Tomiyasu et al. 2006; 2008). Hg pollution has gained global awareness because it is ubiquitous in most environments (AMAP/UNEP 2013) and because its toxicity causes several effects on human health (Guzzi and La Porta 2008). Hg often transforms into highly neurotoxic methyl-mercury (ATSDR 2008) and biomagnifies upward through the food chain in aquatic ecosystems (Kim et al. 2004).

Hg is toxic, but some microorganisms that exhibit resistance are found in systems contaminated with Hg. Various genera of bacteria and fungi have been reported to tolerate Hg (von Canstein et al. 1999 and von Canstein et al. 2002; Huang et al. 1999; Yavuz et al. 2006; Francois et al. 2012). The physicochemical and biological processes leading to the removal and/or sequestration of Hg are bio-remedial strategies proposed to alleviate systems polluted with Hg (Francois et al. 2012; Hussein et al. 2004; Velasquez and Dussan 2009). Pitfalls of non-biological approaches to Hg removal (Hussein et al. 2004) make a microbial-based technology for the detoxification of Hg in polluted systems a better remediation option. The applicability of Hg-resistant microorganisms for the determination of the fate of Hg is of great importance to environmental and public health sciences. Several reports have articulated the use of microorganisms to efficiently remove Hg from contaminated matrixes via biological reduction (von Canstein et al. 1999 and von Canstein et al. 2002), biosorption (Francois et al. 2012; Velasquez and Dussan 2009), bioaccumulation (Velasquez and Dussan 2009), and biosequestration (Francois et al. 2012). Bacterial strains that harbor *merA* genes are known to reduce Hg^2+^ to volatile Hg^0^ (Huang et al. 1999; von Canstein et al. 1999; 2002). Thereby, removing Hg from local environment is achieved by using mercury-resistant microorganisms.

Although yeast cells are generally known to be mediocre in terms of metal biosorption (Voleski 1994), *Yarrowia* species have been reported to exhibit resistance to toxic concentrations of a number of heavy metals (Bankar et al. 2009; Strouhal et al. 2003). Despite many investigations on Hg resistance and detoxification by microorganisms, little is known about Hg resistance of *Yarrowia* species. Based on the available information, neither Hg volatilization nor its biosorption through resting cells has been linked to *Yarrowia* species. The present study sought to find appropriate microbial Hg removal agents using resting and growing Hg-resistant *Yarrowia* yeast cells.

## Materials and methods

### Sampling and chemical reagents

Lagos Lagoon in Nigeria is an estuary water body more than 50 km in length and 3–13 km in width with a surface area of approximately 6400 km^2^. The densely populated city of Lagos spreads along the shore of more than 60 % of the lagoon, which results in the exposure of the water to multifarious contaminants. The highly polluted water empties into the Atlantic Ocean. Composite samples of water and sediment from Lagos Lagoon (06° 27′ 99.7″ N, 003° 22′ 85.1″ E) were collected and analyzed to determine their Hg content (see “Analyses of total mercury” below). HgCl_2_ used in this research was purchased from Sigma-Aldrich Corp. (St. Louis, MO, USA). Stock solutions (100 g l^−1^) of HgCl_2_ were prepared and sterilized at 121 °C. All of the other chemicals used were of analytical reagent grade.

### Isolation, culture conditions, and deposition of isolates

The sediment sample (10 g) was enriched in a sterile starch-casein broth (90 ml) supplemented with 2 mg l^−1^ Hg^2+^. The starch casein broth contained 10 g l^−1^ soluble starch, 2 g l^−1^ K_2_HPO_4_, 2 g l^−1^ KNO_3_, 2 g l^−1^ NaCl, 0.3 g l^−1^ casein, 0.05 g l^−1^ MgSO_4_·7H_2_O, 0.02 g l^−1^ CaCO_3_, and 0.01 g l^−1^ FeSO_4_·7H_2_O, pH 5.5 (approx.). After a 10-day incubation (30 °C, 150 rpm), 100 μl of the culture was spread on a dried surface of sterile Bacto YM broth (Difco, Detroit, MI, USA) solidified with 1.5 % agar (Wako, Osaka, Japan). The YM broth contained 5 g l^−1^ yeast extract, 3 g l^−1^ malt extract, 5 g l^−1^ peptone, and 10 g l^−1^ dextrose. Three days post-inoculation (30 °C), colonies were selected and stored in a water:glycerol (1:1) mixture at −80 °C. The isolated yeast strains have been deposited to Japan Collection of Microorganisms under accession no. JCM 30162 (strain Idd1) and JCM 30163 (strain Idd2).

### Determination of resistance pattern

The isolated yeasts were screened to determine the minimum inhibitory concentration (MIC) of Hg, which is the concentration at which no growth of yeast was observed in the YM broth supplemented with HgCl_2_ (30 °C, 150 rpm, 48 h). The Hg concentrations in the YM broth used for the MIC determination were 2, 4, 8, 16, 32, and 64 mg l^−1^. Two strains of yeast showing resistance to 32 mg l^−1^ were selected and subjected to further analysis.

### Molecular characterization and identification of Hg-resistant yeasts

DNA was isolated and purified using a Wizard DNA isolation kit (Promega Corp., Madison, WI, USA) according to the manufacturer’s instructions. Using universal eukaryotic primer sets (Table S1), nearly full-length 18S ribosomal RNA (rRNA) genes were amplified from the genomic DNA. Each PCR mixture contained 1 μl of DNA template, 5 μl of 10 × Taq DNA polymerase buffer (TaKaRa, Ohtsu, Japan), 4 μl of dNTP (2.5 mmol l^−1^ each; TaKaRa), 0.5 μl of each primer (50 μmol l^−1^), 0.25 μl of Ex-Taq DNA polymerase (TaKaRa), and 38.75 μl of sterile Milli-Q water in a final volume of 50 μl. The PCR protocol consisted of an initial hot incubation (95 °C, 5 min), 30 amplification cycles (denaturation at 95 °C for 45 s, annealing at 55 °C for 1 min, and extension at 72 °C for 2.5 min), and a final extension (72 °C, 7 min) using a Gradient PCR Thermal Cycler Dice (TaKaRa). The 18S rRNA gene amplicons were sequenced using the Applied Biosystems 3130xl Genetic Analyzer and a BigDye Terminator v3.1 cycle sequencing kit (Applied Biosystems, Foster, CA, USA). The sequences were compared to those present in the database using BLAST (N) and aligned using the Muscle 3.7 program. Regions with significant variations were automatically removed using the Gblocks software. The phylogeny of the aligned multiple sequences were analyzed with PhyML v3.0, and a phylogenetic tree was rendered using TreeDyn. The 18S rRNA gene sequences were deposited in GenBank under accession no. AB893170 (strain Idd1) and AB893171 (strain Idd2).

### Determination of mercury removal through biosorption by resting yeast cells

The resuscitated yeast strains (approx. 10^6^ cells ml^−1^ as OD_600_ = 0.1) were starved overnight in Tris–HCl (0.1 mol l^−1^) and re-suspended in sterile Milli-Q water (previously supplemented with 1.0 mg l^−1^ Hg^2+^, final concentration) to prepare resting yeast cells. The yeast cell concentrations in the cell suspensions were determined as previously described (Puranik and Paknikar 1999).

To determine the Hg adsorption capacity of the yeast cells, a sufficient inoculum as prepared above (25 mgdw in 10 ml of deionized water, approx.) was introduced into an Erlenmeyer flask containing 50 ml of minimal medium (MM) supplemented with 6.0, 12.0, 24.0, 48.0, 96.0, or 192.0 mg l^−1^ HgCl_2_, and the culture was incubated (50×*g*; 30 °C). The MM contained 10 g l^−1^ glucose, 2.67 g l^−1^ NH_4_Cl, and 5.35 g l^−1^ Na_2_HPO_4_ in 6 ml of mineral salts solution (0.1 g of CaCl_2_.H_2_O, 10 g of MgSO_4_·7H_2_O, 0.07 g of MnSO_4_·7H_2_O, and 0.04 g of FeSO_4_·7H_2_O in 1000 ml of distilled water). At every 5 min, the quantity of Hg adsorbed by the yeast cells was determined (as described below) after centrifugation (9000×*g*, 20 min). The Hg adsorbed was calculated as $Q=\frac{V\left({C}_O-{C}_e\right)}{m}$, where Q is the specific Hg uptake (mg gdw^−1^), V is the volume of the Hg solution (ml), *C*_*O*_ is the initial Hg concentration in the solution (mg l^−1^), *C*_*e*_ is the final Hg concentration in the solution (mg l^−1^), and *m* is the yeast cell dry weight (gdw). The time at which the Hg-adsorption equilibrium was attained by the resting yeast cells was determined.

### Desorption of Hg from loaded yeast cells

The desorption assay was performed as previously reported (Goyal et al. 2003): aqueous 0.1 mol l^−1^ NaOH, 6.0 mmol l^−1^ KCl, 5.0 mmol l^−1^ CaCl_2_, and 5.0 mmol l^−1^ EDTA were used as the desorption solutions. A cell mass previously loaded with Hg through biosorption was harvested (9000×*g*, 10 min), washed with physiological phosphate buffer, and treated with the desorption solution (50.0 ml) at 30 °C with shaking (100 rpm). The desorption with NaOH was allowed to continue for 20 min, whereas the other solutions were incubated for 8 h due to the disparity between the ionic strength of NaOH and that of the other solutions. The supernatant was separated (9000×*g*, 10 min), and its Hg content was analyzed. Sterile Hg-supplemented broth and broth without Hg inoculated with resting cells were used as the controls, and these were subjected to the same experimental conditions.

### Bioaccumulation of mercury by actively growing yeast cells

The yeast inoculum (1 ml), which was prepared as described above, was inoculated into 100 ml of YM broth supplemented with Hg (0, 3.0, 6.0, 12.0, 24.0, and 48.0 mg l^−1^) in 500-ml Erlenmeyer flasks, and the culture was incubated at 30 °C and 50 *rpm* for 48 h. The growth of the yeast strains was monitored every 6 h based on the turbidity, which was determined by measuring the OD_(600 nm)_ using a UV-visible spectrophotometer. The blank was uninoculated YM broth supplemented with the appropriate Hg concentration. The growing cell of each sample was harvested (10,000×*g*, 10 min), and the supernatant was separated into a new tube. The pellet was washed three times with saline solution (0.8 % NaCl), oven-dried (105 °C) overnight, and weighed. The culture, supernatant, and pellets were analyzed to determine their Hg content as explained below. The controls were the same as those described above.

### Analyses of total mercury

The total mercury amounts in 100 μl of the culture medium and supernatant (obtained from the Hg removal experiments) were determined directly without any pre-treatment using a fully automated thermal vaporization mercury analysis system, i.e., Mercury/MA-3000 (Nippon Instrument Corp., Osaka, Japan). The atomic absorbance of the atomized Hg was measured at a wavelength of 253.7 nm. The instrument was calibrated with standard Hg solution (BDH, Leicestershire, England) at concentrations ranging from 0.1 to 100.0 mg l^−1^. The amount of volatilized Hg was determined by subtracting residual amount of Hg from initial amount in 100 μl of the culture medium.

### Hg^0^ volatilization assay during yeast growth

The volatilization of Hg^2+^ to Hg^0^ during growth of yeast cells was confirmed qualitatively as previously described (Nakamura and Nakahara 1988) and quantitatively in terms of the depletion of Hg. For the qualitative assay, the yeast strains were cultivated in YM broth or YM agar supplemented with 40 μmol l^−1^ HgCl_2_. After 48 h of incubation (30 °C), the colonies on the YM agar plates were harvested and suspended in 100 μl of phosphate buffer (7 mmol l^−1^, pH 7) containing 0.5 mmol l^−1^ EDTA, 0.2 mmol l^−1^ magnesium acetate, and 5 mmol l^−1^ sodium thioglycolate with HgCl_2_ (100 μmol l^−1^) or without HgCl_2_ as the control. For the detection of Hg volatilization using the YM broth culture, 100 μl of the culture was mixed with 100 μmol l^−1^ HgCl_2_ (final concentration). The suspensions were diluted with buffer, described above, to obtain an OD_(600 nm)_ of 0.2. The suspensions were transferred to a microplate, covered with a medical X-ray film (Fujifilm RX-U; Fujifilm Corp., Tokyo, Japan), and cultivated for 8 h at 30 °C in the dark. The foggy areas on the film, which are due to the reduction of the Ag^+^ emulsion of the film by gaseous Hg^0^, were thus interpreted as Hg volatilization. Sterile buffer with and without HgCl_2_ was used as the negative control, whereas *Bacillus megaterium* MB1, which harbors the *merA* gene in its chromosome and expresses Hg^2+^ reductase (Huang et al. 1999), was used as the positive control.

### Statistical analyses

All of the statistical tests were performed using the Prism 5 software program (GraphPad software, San Diego, CA, USA).

### Definition of resting cells and growing cells of the yeasts

“Resting cells” are yeast cells that are harvested after approximately 10^6^ cells ml^−1^ as OD_600_ 0.1 growth was obtained, harvested, and washed twice; the cells are metabolically active but they are not multiplying. While “growing cells” are cells in nutrient medium that are increasing in population and consequently increasing in biomass as time of incubation changes.

## Results

### Sampling, isolation, Hg resistance, and identification of yeast strains

The two Hg-resistant yeast strains were isolated from lagoon sediment that had total mercury concentration of 95 mg kgdw^−1^. The yeast strains grew well in YM medium containing 8 % NaCl and showed resistance, as MIC, to 44.0 mg l^−1^ Hg (220 μM HgCl_2_). Based on their 18S rDNA sequences, the yeast strains were identified as species of *Yarrowia* because they exhibited some relatedness, as determined by their occupation of the same clade in the phylogenetic tree depicted in Fig. S1.

### Hg (II) removal

#### Adsorption equilibrium of Hg (II) by resting yeast cells

**Fig. 1:**
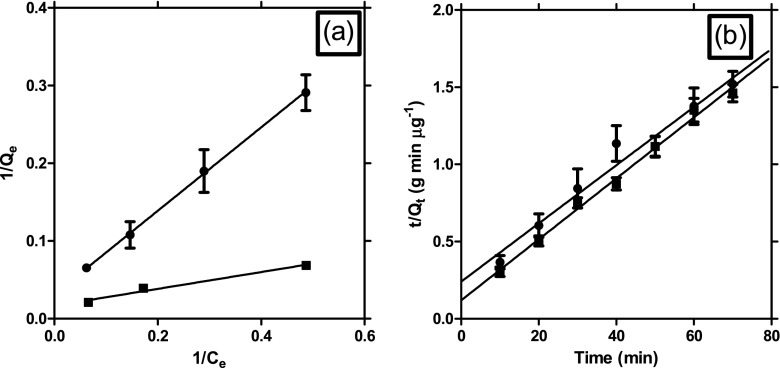
**a** Adsorption equilibrium by Langmuir equation and **b** adsorption kinetics by pseudo-second-order equation of Hg (II) on resting cells of yeast strains Idd1 (*filled cycle*) and Idd2 (*filled square*). The *error bars* represent the standard error of the mean from triplicate experiments

**Fig. 2:**
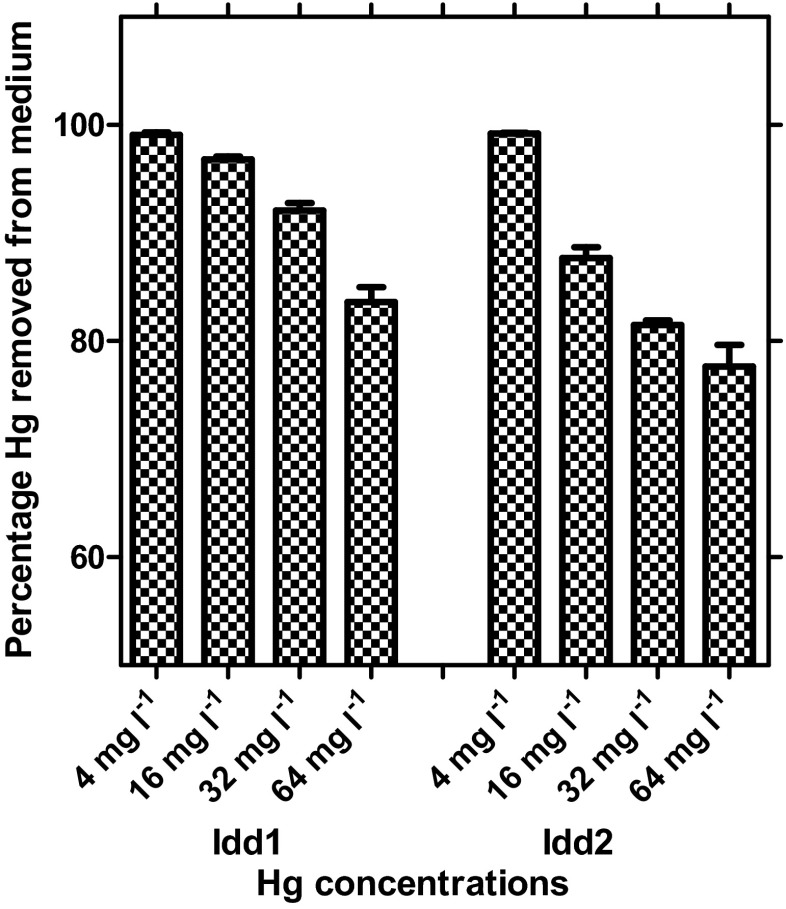
Efficiencies of Hg removal by resting cells of yeast strains Idd1 and Idd2 in medium supplemented with various concentrations of HgCl_2_. The *error bars* represent the standard error of the mean from triplicate experiments

The resting cells of the two yeast strains adsorbed a major fraction of Hg^2+^ within the first 15-min exposure followed by an almost negligible increase after 60 min. The adsorption equilibria were attained 80 min (strain Idd1) and 100 min (strain Idd2) after inoculation. Hg adsorbed by resting yeast cells at equilibrium (Q_e_ mg gdw^−1^) as a function of the concentration of Hg in the solution at equilibrium (*C*_*e*_) (mg l^−1^) was expressed by the Langmuir model equation (${Q}_e=\frac{Q_{\max }{C}_e}{\frac{1}{b}+{C}_e}$) in a linear form as $\frac{1}{Q_e}=\frac{1}{Q_{\max }b}\times \frac{1}{C_e}+\frac{1}{Q_{\max }}$ (see Fig. 1a); where *Q*_max_ (maximum Hg uptake, mg gdw^−1^) and *b* (affinity between Hg^2+^ and the resting yeast cells) are the Langmuir constants. The Langmuir adsorption constants evaluated from the isotherms, as well as their correlation coefficients (*R* ^2^) (≥0.9) show that the Langmuir model provided a fit for the experimental data (Table 1). The Hg adsorption data indicates that strain Idd2 is characterized by a higher maximum Hg^2+^ (59 mg gdw^−1^) than strain Idd1 (32 mg gdw^−1^). The Langmuir affinity (saturation) constant (1/*b*) was marginally higher in strain Idd1 (17 mg^−1^) than Idd2 (6.5 mg^−1^). Mercury removal percentage of the resting cells is represented in Fig. 2, where more than 99 % of available Hg^2+^ was removed at low metal concentration (4 mg l^−1^). At high metal concentration (64 mg l^−1^), however, resting cells of Idd1 removed Hg^2+^ more efficiently (84 ± 2 %) than Idd2 (78 ± 4 %).

**Table 1:** Hg^2+^ biosorption kinetics obtained from Langmuir isotherm and pseudo-second-order rate equation for resting cells of yeast strains

Kinetic models	Parameters	Yeast strains	
		*Yarrowia* (Idd1)	*Yarrowia* (Idd2)
Langmuir equation	*Q*_max_ (mg gdw^−1^)	32.0	59.0
	1/*b* (mg l^−1^)	17.0	6.5
	*R* ^2^	0.91	0.98
	*P*	<0.0001	<0.0001
Pseudo second order	*k*_2_ (mg^−1^ gdw min^−1^)	1.5	3.3
	*Q*_*e*_ (mg gdw^−1^)	53.0	51.0
	*R* ^2^	0.86	0.96
	*P*	<0.0001	<0.0001

The pseudo-first-order rate equation, *In*(*Q*_*e*_ − *Q*_*t*_) = *InQ*_*e*_ − *k*_1_ *t*, and the pseudo-second-order rate equation, $\frac{t}{Q_t}=\frac{t}{Q_e}+\frac{1}{k_2{Q^2}_8}$, were represented in their linear forms. In these equations, *Q*_*t*_ is the amount of Hg^2+^ adsorbed (mg gdw^−1^) at any given time *t* (min), *Q*_*e*_ is the amount of Hg^2+^ adsorbed (mg gdw^−1^) at equilibrium, and *k*_1_ and *k*_2_ are the rate constants of the pseudo-first-order reaction and the pseudo-second-order reaction for biosorption, respectively. For the analysis of the applicability of the pseudo-first-order kinetic model, a plot of *In* (*Q*_*e*_ − *Q*_*t*_) as a function of *t* did not yield straight lines (Fig. S1). However, a plot of *t*/*Q*_*t*_ as a function of *t* resulted in straight lines, thereby indicating the applicability of the pseudo-second-order kinetic model (Fig. 1b). The calculated values of *Q*_*e*_ and *k*_2_ are shown in Table 1. The adsorption of Hg by the yeasts follows pseudo-second-order kinetics, as inferred by the high values of *R* ^2^.

### Desorption of Hg from resting yeast cells

The desorption of Hg from Hg-adsorbed resting yeast cells is shown for strain Idd1 and strain Idd2 (Table 2). Solutions of CaCl_2_ (5.0 mM) and EDTA (5.0 mM) desorbed 68 and 48 % of the loaded Hg (II), respectively, from the resting yeast cells of strain Idd2. Hg adhered more strongly to strain Idd1, as indicated by the poorer performance of the desorbing agents (EDTA desorbs 29 % of the adsorbed Hg from the resting cell mass). In addition, the NaOH (0.1 M) solution dislodged a negligible portion (0.02 % from strain Idd1 and 0.03 % from strain Idd2).

**Table 2:** Desorption of Hg^2+^ from yeast strains Idd1 and Idd2 using selected desorbents

	Mercury desorbed from yeast cells (%)	
Desorption solution	Idd1	Idd2
EDTA	29.0 (±1.4)	24.0 (±6.0)
KCl	3.2 (±0.53)	11.0 (±1.0)
CaCl_2_	7.0 (±1.6)	33.0 (±18.0)
NaOH	2.3 (±0.2)	1.6 (±0.085)

Desorption conditions were EDTA (5 mmol l^−1^, 8 h), KCl (5 mmol l^−1^, 8 h), CaCl_2_ (5 mmol l^−1^, 8 h), and NaOH (0.1 mol l^−1^, 20 min). The Hg^2+^ quantities adsorbed by Idd1 and Idd2 were 55.0 (±2.4) mg gdw^−1^, and 49.0 (±3.2) mg gdw^−1^, respectively. Desorption reactions were conducted with shaking (50×*g*) at a temperature of 30 °C. The values indicate the mean (±SD) from triplicate experiments

### Hg volatilization and Hg bioaccumulation by actively growing yeast cells

Hg^2+^ removal was recorded for each growing strain under the ambient pH (5.0–6.5) and temperature (30 ± 2 °C), conditions prevalent in Lagos Lagoon. The yeast strains grew without a lag phase in medium supplemented with 8.0 mg l^−1^ Hg^2+^ (and lower Hg^2+^ concentrations), and the characteristic growth pattern observed was similar to that obtained in medium without Hg^2+^ supplementation. However, at higher Hg concentrations (≥16.0 mg l^−1^), a lag phase (≥12 h) preceded the spontaneous growth of both strains. Adsorption and volatilization of Hg were observed during the lag phase. Hg bioaccumulation began during the exponential growth phase and increased during the stationary phase. The mass balance of Hg^2+^ in the batch culture system was presented in Table 3. Volatilization due to the release of Hg^0^ from the growing yeast cultures was observed based on the development of foggy areas on the X-ray film (Fig. S3).

**Table 3:** Mercury removal by yeast strains Idd1 and Idd2 during growth in medium supplemented with 32 mg Hg^2+^ per liter

Yeast strains	Mercury remaining in medium^a^ (%)	Mercury accumulated onto growing yeast cells^a^ (%)	Mercury volatilized by growing yeast cells^a^ (%)
Idd1	58.0 ± 2.9	16.0 ± 10.8	27.0 ± 13.2
Idd2	63.0 ± 7.8	18.0 ± 14.0	18.0 ± 6.3

^a^Total mercury is expressed as percentages of the initial Hg content in the medium upon inoculation. Values represent the mean (±standard deviation) from triplicate experiments

## Discussion

Reckless waste disposal of industrial and artisanal activities are the major anthropogenic sources through which Hg contaminates the environment throughout the world (AMAP/UNEP 2013). The total Hg concentration in the sediment is higher than those reported in other climes (Tomiyasu et al. 2008) and alarmingly above the permissive level in the environment. Severe hazards to public health and estuarine ecosystems due to Hg toxicity are imminent because there is no known mitigation plan to salvage the continual pollution of the water bodies with Hg-laden effluents. The isolation of highly Hg-resistant yeast strains from the sediment in a mercury contaminated lagoon is an indication of the contamination of the estuarine water-course with mercuric ions. As a bio-indicator of mercuric pollution, the autochthonous yeast strains in the lagoon must have molecular alterations to evolve the high Hg resistance.

The equilibrium distribution of mercuric ions between the aqueous and solid phases is represented by the biosorption rate and the maximum biosorption capacity, which is commonly described by the Langmuir isotherm model (Febrianto et al. 2009). The resting cells of both strains adsorb Hg^2+^ from solution at an almost equal rate. The high *Q*_max_ observed in Hg adsorption indicates that resting cells of strain Idd2 has a higher number of sites available for Hg binding and belongs to a single-type phenomenon with no interactions between the adsorbed Hg. The lower Langmuir saturation constant of strain Idd2 than that of strain Idd1 indicates the adsorption affinity of the resting yeast strain Idd2 for mercuric ions is higher than strain Idd1. However, certain inconsistencies in the saturation capacities (*Q*_max_) of Langmuir equation, and Hg^2+^ uptake at equilibrium (*Q*_*e*_) were observed. The rather higher *Q*_*e*_ (53.0 mg gdw^−1^) than predicted *Q*_max_ (32.0 mg gdw^−1^) recorded for strain Idd1 may be associated with the surface functional groups, and effects of several factors including number of binding sites, chemical state of the sites, and binding strength of the sites, as previously discussed by Febrianto et al. (2009).

The kinetics of Hg^2+^ biosorption on resting yeast cells of *Yarrowia* spp. was determined using the pseudo-second-order model based on solid-phase adsorption (Febrianto et al. 2009) (Fig. 1b and Table 1). The results suggest that chemisorption may be the rate-limiting step that controls the uptake of Hg^2+^ from aquatic systems. The rate-control may vary during the course of Hg^2+^ biosorption (Febrianto et al. 2009). An external surface mass transfer or a film diffusion process may control the early stages of the Hg^2+^ biosorption process onto resting cells of the yeast strains. This stage may be followed by a chemical reaction or constant rate stage and then by a diffusion stage in which the biosorption process slows down considerably (Djeribi and Hamdaoui 2008). It is noteworthy that good fitting model does not necessarily illustrate the nature of real adsorption between the resting cells and Hg^2+^ due to dynamic characteristics of all living cells unlike dead cells.

Desorption upon treatment with calcium and potassium showed a lower desorption performance of strain Idd1 than that of strain Idd2. This negates the potential reusability of strain Idd1 in the treatment of mercury-laden wastewater. From these data, the resting cells of strain Idd2 would be a better option in the treatment of wastewater laden with mercuric ions, since the adsorbed mercuric ions could be readily recovered from the resting cells. The results of Hg desorption from the resting cell masses may indicate that salt cations, particularly Ca^2+^, in estuarine water can readily desorb Hg from the resting cells of strain Idd2 (Table 2), and thereby spread the toxic metal to other parts of the ecosystem. However, strain Idd2 would be of great value for the treatment of industrial wastewater due to its reusability and the potential ability to recover Hg from the resting yeast cells. The poor desorption potential of strain Idd1 coupled with its higher *Q*_max_ demonstrates that the organism may act as an Hg (II) sink in the limnetic zone. However, strain Idd1 would not have predominance over strain Idd2 in recovery of Hg and reusability of the yeast cells for Hg removal in a bioreactor system.

Hg^2+^ reduction to Hg^0^ and Hg^0^ volatilization (Table 3 and Fig. S3) were likely triggered by sulfhydryl compounds present in the biomolecules produced by the growing yeast strains in response to Hg toxicity. Larger amount of mercury was reduced and volatilized by the growing strain Idd1 than the growing strain Idd2. Recently, Rao et al. (2013) reported the role played by strong reducing agents in facilitating efficient metal removal by *Yarrowia lipolytica*. It has been reported that mercury is transported across the vacuolar membrane to become localized in the vacuoles of *Saccharomyces cerevisiae* (Diffels et al. 2006). Certain proteins (Strouhal et al. 2003) and/or interactions with sulfhydryl-containing residues have been hypothesized to be responsible for Hg bioaccumulation in microorganisms (Hansen et al. 2006; Park and Park 2007; Pulido and Parrish 2003). Concrete evidence of the actual organic biomolecules involved in Hg reduction and transportation by *Yarrowia* spp. remains to be obtained.

Based on the results described above, characteristics of mercury removal by the resting and growing cells of yeast strains *Yarrowia* sp. Idd1 and Idd2 isolated in this study were observed. The resting cells of yeast strain Idd2 would be applicable to Hg removal and recovery from mercury-laden water as a more appropriate and reusable bio-adsorbent. In contrast, the growing cells of yeast strain Idd1 would be applicable as an appropriate Hg^2+^ bio-reduction and volatilization agent. Therefore, the most appropriate Hg removal method should be selected according to the removal characteristics of resting cells and growing cells of the yeast strains. However, further studies are needed to understand detailed physiological Hg removal mechanisms of the resting and growing cells of yeast strains, by analyzing biomolecules involved in the Hg reduction and adsorption.

## Electronic supplementary material

Below is the link to the electronic supplementary material.

ESM 1 (DOC 443 kb)
