# Supplementary material for: Mercury bioremoval by Yarrowia strains isolated from sediments of mercury-polluted estuarine water
Source: Appl Microbiol Biotechnol. 2014 Dec 18;99(8):3651–7. doi: 10.1007/s00253-014-6279-1 (PMC4375293; doi:10.1007/s00253-014-6279-1)
Supplement: Supplementary file 1 — (DOC 443 kb) [file 253_2014_6279_MOESM1_ESM.doc]

**Supplementary materials**

**Journal name: Applied Microbiology and Biotechnology**

**Article title:**

**Mercury-Bioremoval by　*Yarrowia* Strains Isolated from Sediments of Mercury Polluted Estuarine Water**

**Author names:**

**Ganiyu Oladunjoye Oyetibo ^a,b^, Shakirat Titilayo Ishola ^b^, Wakako Ikeda-Ohtsubo ^a^, Keisuke Miyauchi ^a^, Matthew Olusoji Ilori ^b^, Ginro Endo ^a*^**

**Affiliations:**

**^a^ Department of Civil and Environmental Engineering, Faculty of Engineering, Tohoku Gakuin University, 1-13-1 Chuo, Tagajo, Miyagi 985-8537 Japan.**

**^b^ Department of Microbiology, Faculty of Science, University of Lagos, Akoka, Yaba, Lagos, Nigeria.**

**^*^ E-mail address of the corresponding author:**

**Ginro Endo:** [**gendo@mail.tohoku-gakuin.ac.jp**](mailto:gendo@mail.tohoku-gakuin.ac.jp)

**Supplementary documents**

**Table S1**: List of primers used in amplification of 18S rRNA gene

| Primer | Sequence (5' - 3') | Product size | Reference |
| --- | --- | --- | --- |
| EukA | AACCTGGTTGATCCTGCCAGT | 1800 | Medlin et al., (1988) |
| EukB | TGATCCTTCTGCAGGTTCACCTAC |  | Medlin et al., (1988) |
| E528F | CGGTAATTCCAGCTCC | 1000-1300 | Edgcomb et al. (2002) |
| nu-SSU-1196 | TCTGGACCTGGTGAGTTTCC | 700-800 | Borneman and Hartin (2000) |

**References**

Borneman, J., Hartin, R.J., 2000. PCR primers that amplify fungi rRNA genes from environmental samples. Appl Environ Microbiol 66, 4356-4360.

Edgcomb, V.P., Kysela, D.T., Teske, A., de Vera Gomez, A., Sogin, M.L., 2002. Benthic eukaryotic diversity in the Guaymas Basin hydrothermal vent environment. Proc Nat Acad Sci USA 99, 7658—7662.

Medlin, L., Elwood, H.J., Stickel, S., Sogin, M.L., 1988. The characterization of enzymatically amplified eukaryotic 16S-like rRNA-coding regions. Gene 71, 491—499

**Fig. S1**: Phylogenetic tree based on 18S rRNA gene sequences showing the relationship between the yeast strains (*Idd1* and *Idd2*) and the most closely related species. The phylogeny of aligned multiple sequences were analysed with PhyML v3.0, and phylogenetic tree was rendered by TreeDyn ([http://www.phylogeny.fr/version2_cgi/](http://www.phylogeny.fr/version2_cgi/ ) ). The scale bar represents substitution per nucleotide (showing 1 bp differs at every 100 bp sequences). *Candida khmerensis* (AB158655) was used as out-group for tree rooting.

**Fig. S2**: Kinetic fitting by pseudo-first-order equation for uptake of Hg(II) on wet cells of live yeast strains *Idd1* (filled cycle), and *Idd2* (filled square). R^2^ for *Idd1* is 0.7131, and *Idd2* is 0.7392.


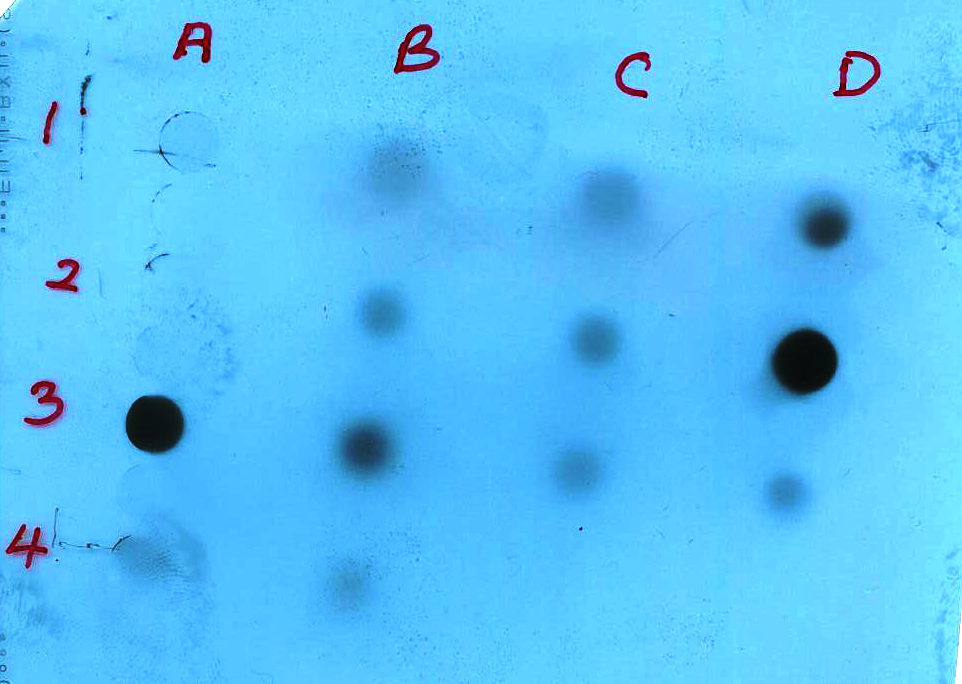


**Fig. S3**: Semiquantitative volatilisation of Hg^2+^ by yeast strains during growth in medium supplemented with HgCl_2_ using X-ray film. The dark zones show degree of foggy formation due to displacement of Ag^+^ of the film by Hg^0^ liberated from microplate wells. Colonies of *Bacillus megaterium* MB1 in 100 µmol l^-1^ HgCl_2_ buffer as positive control (3A), *Idd2* colonies in buffer without HgCl_2_ (1B), *Idd2* colonies in buffer with HgCl_2_ (2B), *Idd2* supernatant with HgCl_2_ (3B), *Idd2* supernatant without HgCl_2_ (4B), *Idd1* colonies in buffer without HgCl_2_ (1C), *Idd1* colonies in buffer with HgCl_2_ (2C), *Idd1* supernatant with HgCl_2_ (3C), *Idd1* supernatant without HgCl_2_ (4C), *Idd1* broth culture with HgCl_2_ (1D), *Idd2* broth culture with HgCl_2_ (2D), *Idd2* broth culture without HgCl_2_ (3D), *Idd1* broth culture without HgCl_2_ (4D), negative controls are buffer only (1A), Buffer with Hg (2A), and YM medium with HgCl_2_ (4A).
